# Supplementary figures and images for: Reconciling high-throughput gene essentiality data with metabolic network reconstructions
Source: PLoS Comput Biol. 2019 Apr 11;15(4):e1006507. doi: 10.1371/journal.pcbi.1006507 (PMC6478342; doi:10.1371/journal.pcbi.1006507)

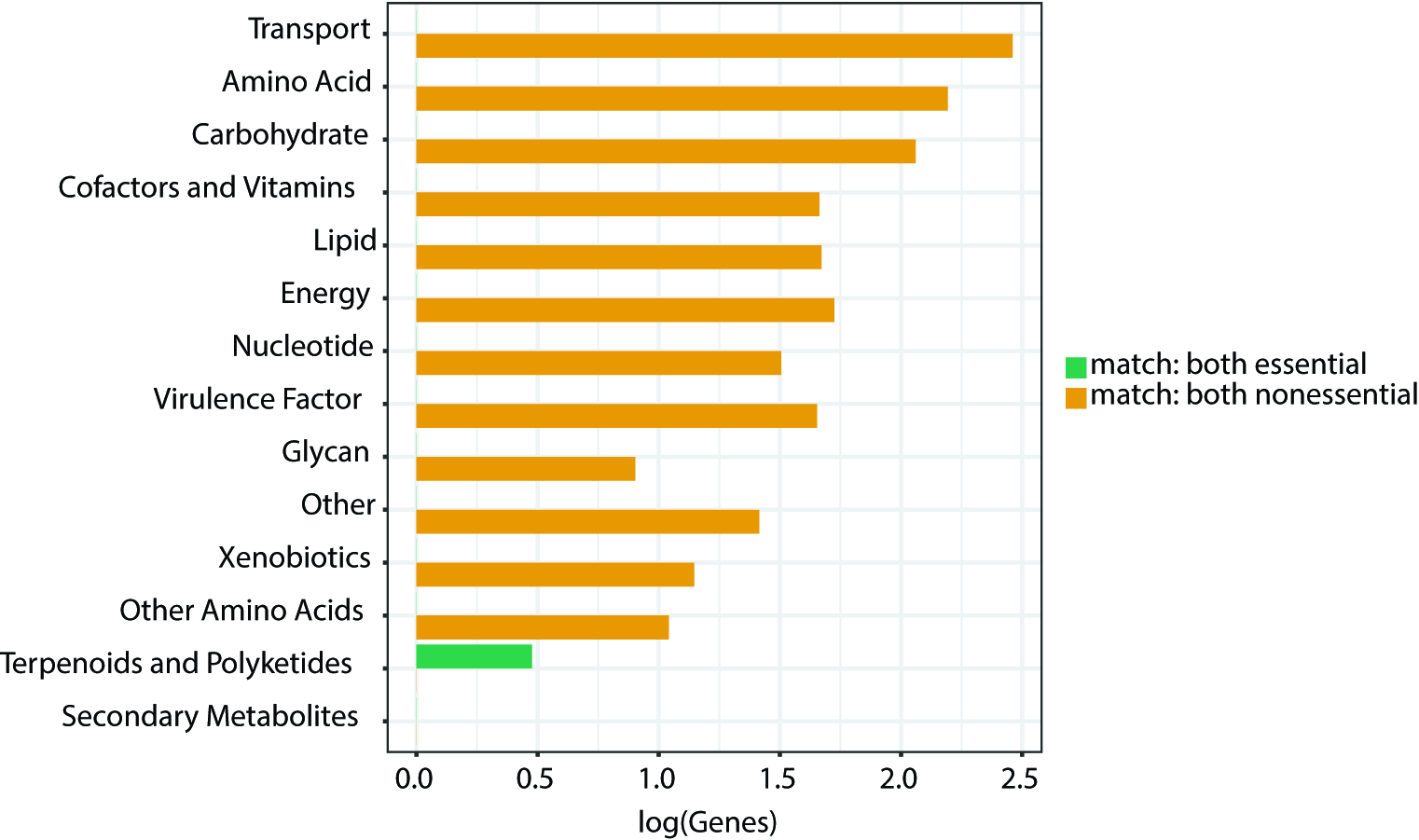

Supplement: S1 Fig — (TIF) [file pcbi.1006507.s012.tif]

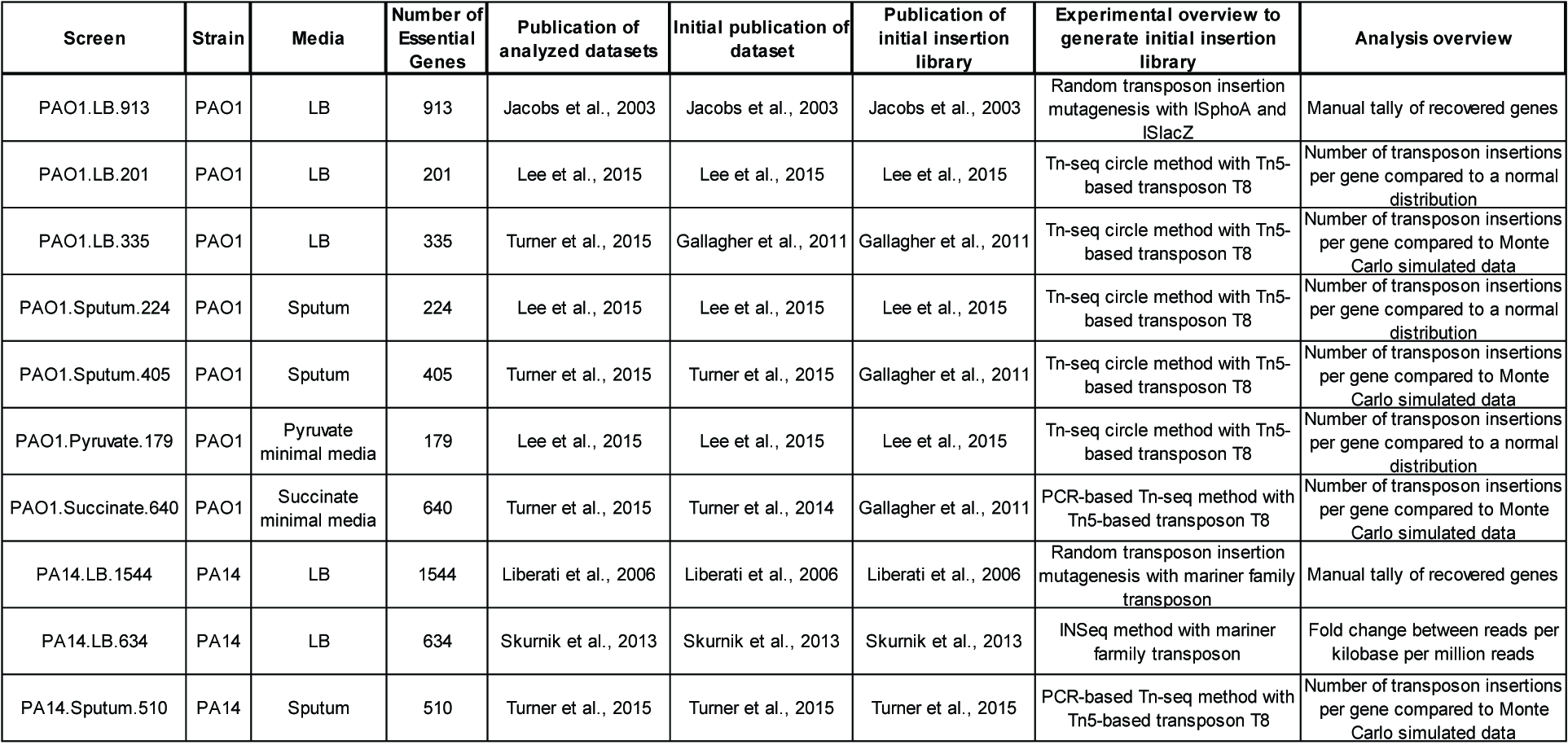

Supplement: S1 Table — (TIF) [file pcbi.1006507.s013.tif]

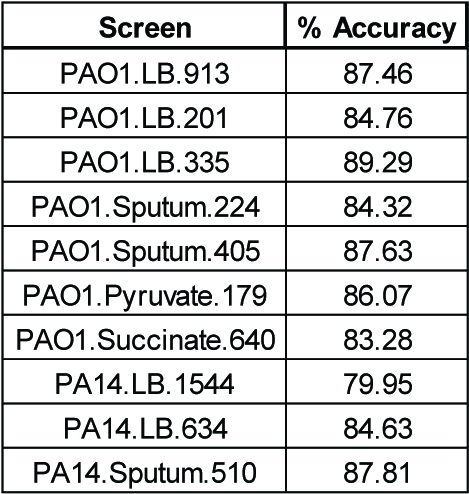

Supplement: S2 Table — (TIF) [file pcbi.1006507.s014.tif]

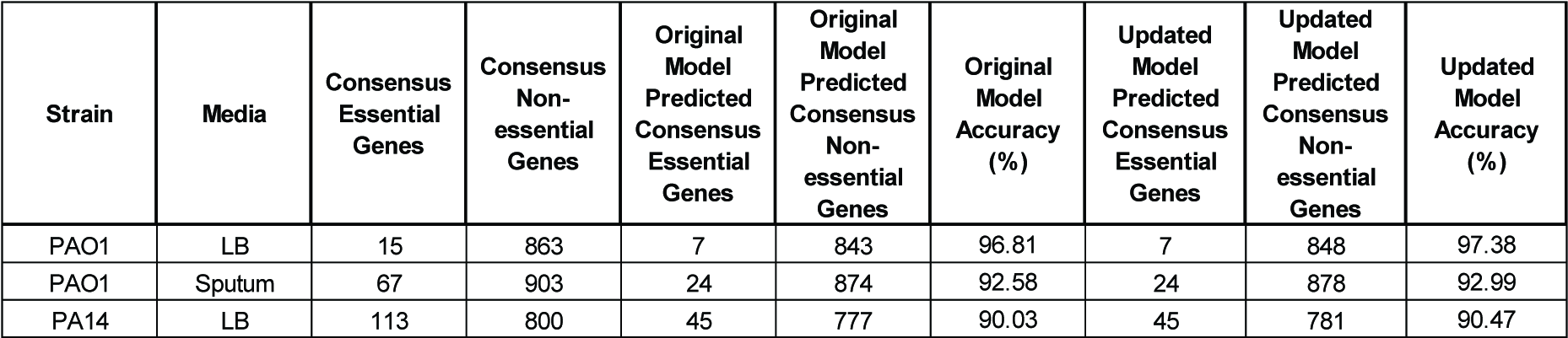

Supplement: S3 Table — (TIF) [file pcbi.1006507.s015.tif]
